# Economic burden of eating disorders in South Korea

**DOI:** 10.1186/s40337-021-00385-w

**Published:** 2021-03-04

**Authors:** Sang Min Lee, Minha Hong, Saengryeol Park, Won Sub Kang, In-Hwan Oh

**Affiliations:** 1 Department of Psychiatry, Kyung Hee University Hospital, Kyung Hee University College of Medicine, 1 Hoegi-Dong, Dongdaemug-Gu, Seoul, 130-701 South Korea; 2 Department of Psychiatry, Myongji Hospital, Hanyang University College of Medicine, 55 Hwasu-ro 14 beon-gil, Deogyang-gu, Goyang, 10475 South Korea; 3 Department of Physical Education, College of Education, Chonnam National University, 77 Yongbong-ro, Buk-gu, Gwangju, 61186 South Korea; 4 Department of Preventive Medicine, Kyung Hee University School of Medicine, 1 Hoegi-Dong, Dongdaemug-Gu, Seoul, 130-701 South Korea

**Keywords:** Eating disorder, Prevalence, Burden of disease, Bulimia nervosa, Anorexia nervosa

## Abstract

**Background:**

Few studies have investigated the epidemiology of eating disorders using national representative data. In this study, we investigated the treatment prevalence and economic burden of eating disorders in South Korea.

**Methods:**

The aim of this study was to estimate the treatment prevalence and the medical expenditure of diagnosed eating disorders (ICD F50.x) in South Korea between 2010 and 2015. We also examined the economic costs of eating disorders, including the direct medical cost, direct non-medical costs, and indirect costs, in order to calculate the economic burden of such disorders.

**Results:**

The total treatment prevalence of eating disorders in South Korea was 12.02 people (per 100,000) in 2010, and 13.28 in 2015. The cost of medical expenditures due to eating disorders increased from USD 1229724 in 2010 to USD 1843706 in 2015. The total economic cost of eating disorders was USD 5455626 in 2015. In 2015, the economic cost and prevalence of eating disorders was the highest in the 20–29 age group.

**Conclusions:**

The results showed the eating disorders are insufficiently managed in the medical insurance system. Further research is therefore warranted to better understand the economic burdens of each type of eating disorder.

## Plain English summary

This article is the result of estimating the overall medical expenditures due to eating disorders in South Korea, a country that has introduced the National Health Insurance system, the prevalence rate based on this, and further the economic burden. It is a data that can grasp the status and actual condition of medical expenses due to eating disorders, and can be the basis for appropriate distribution of medical expenses and policy-making process in the future.

## Background

There is evidence that eating disorders are increasing worldwide, and that they affect approximately 2% of the world’s population [1, 2]. Eating disorders may occur at a relatively young age, often beginning between 10 and 20 years of age [3], and may be chronic, lifelong conditions that are associated with various physical and psychiatric components [4, 5]. They are also one of the most common adolescent chronic disorders [6, 7], and friends and family often become informal long-term caregivers [8]. Among mental illnesses, eating disorders have the highest lifetime mortality rate (up to 20%); the mortality rate among women with eating disorders is twelve times higher than it is for unaffected women [9, 10]. When compared with the general population, people with eating disorders have nearly double the mortality rate of those who are unaffected [11].

A study of patients with eating disorders in the United States found that the majority of patients did not seek treatment for the eating disorder itself [5]. It was similar phenomenon in Asia that the proportion of patients within the healthcare setting is low [12, 13]. Even when eating disorders are treated, medication has limited efficacy and, in general, more than half the patients with anorexia and bulimia nervosa do not recover fully [1, 6]. One in four people with anorexia nervosa develops long-term impairment in social functioning and employment, to the extent that they cannot be gainfully employed. The quality of life for patients with eating disorders deteriorates more than it does for patients with symptomatic coronary heart disease or major depression, and the duration of illness tends to be longer [14].

Treatment guidelines recommend the active involvement of family members in the treatment of eating disorders [15]. Patients with severe and long-lasting anorexia nervosa are highly dependent on their families, creating a subsequently high caregiving burden [16]. The socio-economic burden and costs of anorexia nervosa and bulimia nervosa are similar to those of anxiety disorders and depression [6], as quantified by the Global Burden of Disease Study conducted in 2013 [17].

Studies have been conducted in Europe to estimate the size and cost of eating disorders, but most have included only anorexia nervosa and bulimia nervosa; this led to a gross underestimation of the problem, because binge eating and unspecified eating disorders are in fact the most commonly occurring disorders [18]. Those studies also did not include key resource items: the cost of lost productivity for the entire family, and indirect costs due to reduced length of life and health [6, 18].

Only some recently published studies presented a partial aspect of epidemiology of eating disorders in Asia [12, 13, 19]. There have been very few studies of epidemiology of eating disorders completed in South Korea. Lee et al. published a psychiatric epidemiology of major disorders using DSM-III criteria [20, 21]. Cho et al. reported that the lifetime prevalence of eating disorders using DSM-IV criteria in Korea was 0.2% [22].

Globally, several studies have systematically reviewed the disease burden of eating disorders. Extant studies of eating disorders tend to have poor data representation due to the lack of large-scale population based studies and the inconsistencies of studies [17]. This study analyzed the healthcare costs of anorexia nervosa, bulimia nervosa, and other eating disorders, such as binge eating disorder and eating disorders not otherwise specified, over a six-year period. Using representative health statistics and health insurance data from 2010 to 2015, we attempted to estimate the national burden and economic costs of eating disorders on medical care utilization and to explore the characteristics of this burden with respect to gender and age groups.

## Methods

### Data sources

This study utilized two government data sources for its analysis. The prevalence rates and medical expenditure of eating disorders were calculated using data from the Health Insurance Review & Assessment Service (HIRA). The database provided records of patient numbers and specified outpatient, inpatient, and hospitalization days by gender. The economic cost of eating disorders was derived from the data of the National Health Insurance Services (NHIS), which is the single insurer in South Korea [23]. The NHIS provides medical costs based on the medical utilization records from the National Health Information Database (NHID). Data from January 1, 2020 to December 31, 2015 were collected from both HIRA and NHIS. Population statistics were adopted from the Korean Statistical Information Service (KOSIS). Average currency rates per year were adopted from the Bank of Korea (http://ecos.bok.or.kr) to convert the Korean Won to US dollars (USD). The data supporting this study’s findings are available on request from the corresponding author, but are not publicly available due to privacy or ethical restrictions.

### Case definition

Eating disorders (F50) were defined using the International Classification of Diseases, Tenth Revision (ICD-10) [24]. For estimation of the economic burden, eating disorders were as: anorexia nervosa (F50.0); bulimia nervosa (F50.2); and other eating disorders (OED) (F50.1–F50.9). OED included atypical anorexia nervosa (F50.1); atypical bulimia nervosa (F50.3); overeating associated with other psychological disturbances (F50.4); vomiting associated with other psychological disturbances (F50.5); other eating disorders (F50.8); and unspecified eating disorder (F50.9).

### Treatment prevalence rates of eating disorders

The treatment prevalence rates of eating disorders from 2010 to 2015 were estimated using the number of cases from HIRA Service. The number of cases was divided by the total population and then multiplied by 100,000.

### Estimation of the economic burden of eating disorders

The present study estimated the medical expenditure and economic cost of eating disorders (anorexia nervosa, bulimia nervosa, OED) using data from HIRA and NHIS. Medical expenditure was determined by the HIRA data regarding expenditures from both the national insurance service and patients. Economic cost, both direct and indirect, was estimated using a prevalence-based approach from NHIS data.

Direct costs included the total costs associated with medical treatment, transportation, and caregivers. Medical costs included non-covered care costs, insured and non-insured costs, and drug costs. Direct non-medical costs included transportation costs and caregiver costs. Transportation costs associated with eating disorders were defined as the products of the number of outpatient visits and hospitalizations with the average roundtrip transportation costs. The average roundtrip transportation costs were 4.34 USD per outpatient visit and 46.70 USD per hospitalization according to Korean Health Panel data. The time spent for an outpatient visit was estimated as one-third of the cost for an inpatient visit for determination of outpatient caregiver costs. Also, caregiver costs were calculated using data from the Korea Patient Helper Society.

Indirect costs-2 was estimated to explain productivity loss caused by the absence from work for hospital admissions or outpatient visits. Indirect costs-2 was included in the total costs. For sensitivity purposes, indirect costs-1 was estimated by considering lost productivity. Productivity lost was defined as the loss of ones’ time due to medical care. To estimate the productivity lost we used time spent traveling to hospital and waiting for treatment and multiplied the average time spent by the average daily wage. For example, when a patient took the day off due to hospitalization, it was considered as the loss of one day’s income. In case of an outpatient visit, it was considered as the loss of one-third of daily income. Data were not available for those under 20 years old as they are too young to work. Indirect costs-1 was not included in the total costs. Total economic cost was taken as the sum of direct and indirect costs.

All analyses were performed using SAS (ver. 9.4; SAS institute, Cary, NC, USA).

### Ethics statement

Ethical review was obtained by a University review board (IRB No. KHSIRB-19-354 (EA)). Informed consent was exempted due to the public nature of the NHIS data. The information is gathered by ID number, it is not identifiable.

## Results

The current study investigated the treatment prevalence rates of eating disorders and patients’ use of medical care between 2010 and 2015, in addition to evaluating the economic burden of eating disorders in Korea in 2015.

**Fig. 1:**
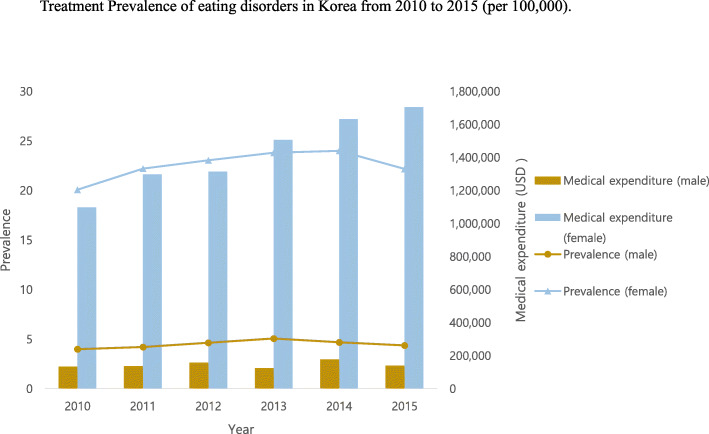
Treatment Prevalence of eating disorders in Korea from 2010 to 2015 (per 100,000)

The results of this study showed that the treatment prevalence rates of eating disorders tended to increase from 2010 to 2013 and then decreased slightly from 2014 to 2015 (Table 1 and Fig. 1). The medical expenditure of eating disorders consistently increased from USD 1229724 in 2010 to USD 1843706 in 2015. Cases of bulimia nervosa increased from 2010 to 2015. In addition, a gender differential was observed in the economic burden of eating disorders from 2010 to 2015; the discrepancy was higher in female patients than in to male patients.

**Table 1:** Treatment Prevalence of eating disorders in Korea from 2010 to 2015 by gender (per 100,000)

	Eating disorders Number of patients Prevalence			Anorexia nervosa Number of patients Prevalence			Bulimia nervosa Number of patients Prevalence			Other eating disorders Number of patients Prevalence			Medical expenditure of eating disorders		
Year	Sub total	Male	Female	Sub total	Male	Female	Sub total	Male	Female	Sub total	Male	Female	Male	Female	Total cost
2010	6074	1010	5064	1511	376	1135	1399	72	1327	3366	572	2794	131,770	1,097,954	1,229,724
	12.02	3.99	20.09	2.99	1.49	4.50	2.77	0.28	5.26	6.66	2.26	11.08			
2011	6694	1070	5624	1570	405	1165	1440	74	1366	3888	607	3281	135,824	1,298,591	1,434,415
	13.19	4.21	22.21	3.09	1.59	4.60	2.84	0.29	5.39	7.66	2.39	12.95			
2012	7052	1187	5865	1534	369	1165	1600	92	1508	4151	754	3397	155,809	1,314,500	1,470,310
	13.84	4.65	23.05	3.01	1.45	4.58	3.14	0.36	5.93	8.15	2.96	13.35			
2013	7388	1301	6087	1905	478	1427	1597	111	1486	4099	727	3372	123,037	1,506,356	1,629,394
	14.45	5.08	23.82	3.72	1.87	5.58	3.12	0.43	5.82	8.02	2.84	13.20			
2014	7364	1204	6160	1793	457	1336	1681	93	1588	4110	680	3430	175,329	1,631,515	1,806,843
	14.35	4.69	24.01	3.49	1.78	5.21	3.28	0.36	6.19	8.01	2.65	13.37			
2015	6845	1129	5716	1604	397	1207	1832	123	1709	3614	630	2984	138,939	1,704,767	1,843,706
	13.28	4.38	22.18	3.11	1.54	4.68	3.56	0.48	6.63	7.01	2.45	11.58			

*Note*. Data sources from Healthcare Bigdata Hub (https://opendata.hira.or.kr/) and Korean Statistical Information Service (KOSIS); size of population = 50,515,666 (female 25,205,281; 2010); 50,734,284 (female 25,327,350; 2011); 50,948,272 (female 25,444,212; 2012); 51,141,463 (female 25,553,127; 2013); 51,327,916 (female 25,658,620; 2014); 51,529,338 (female 25,771,152; 2015); Exchange rate US dollar: 1 Korean won = 1132 US dollar (2015); 1053 (2014); 1095 (2013); 1127 (2012); 1108 (2011); 1156 (2010); from the Bank of Korea (http://ecos.bok.or.kr/)

Table 2 shows the medical care use of eating disorders, including anorexia nervosa, bulimia nervosa, and OED, from 2010 to 2015. There was an inconsistent increase in the number of outpatient visits of patients afflicted with different types of eating disorders. The number of inpatient admissions decreased for patients with bulimia nervosa but increased in the cases with anorexia nervosa and OED. Hospitalization days per patient inconsistently decreased in anorexia nervosa and bulimia nervosa, but increased in OED.

**Table 2:** Patient’s medical care use for eating disorders from 2010 to 2015

Year	Eating disorders			Anorexia nervosa			Bulimia nervosa			Other eating disorders		
	Number of outpatient visits (per patient)	Number of inpatient admissions (per patient)	Hospitalization days (per patient)	Number of outpatient visits (per patient)	Number of inpatient admissions (per patient)	Hospitalization days (per patient)	Number of outpatient visits (per patient)	Number of inpatient admissions (per patient)	Hospitalization days (per patient)	Number of outpatient visits (per patient)	Number of inpatient admissions (per patient)	Hospitalization days (per patient)
2010	3.27	1.72	31.16	2.43	1.51	28.32	4.03	1.67	37.62	3.19	1.33	17.76
2011	3.07	1.80	30.40	2.30	1.93	34.26	4.20	1.77	32.95	2.92	1.13	14.65
2012	3.54	1.79	30.48	2.73	1.97	35.17	4.57	1.63	29.07	3.25	1.24	17.58
2013	3.49	1.82	29.83	2.49	1.90	35.11	4.44	1.78	22.76	3.42	1.40	17.97
2014	3.51	1.71	28.69	2.71	1.65	29.42	4.16	1.66	25.78	3.15	1.34	17.14
2015	3.86	1.65	27.01	2.86	1.62	27.26	4.52	1.63	28.25	3.40	1.47	22.05

*Note*. Data source from Healthcare Bigdata Hub (https://opendata.hira.or.kr/)

Table 3 shows the economic burden of eating disorders including anorexia nervosa, bulimia nervosa, and OED by gender in 2015. The economic cost of eating disorders was 5,455,626 USD. Total costs were approximately 6 times higher in female patients than male patients. Direct costs were higher than indirect costs-2 in all types of eating disorders. OED were the highest contributor to the economic burden among anorexia nervosa, bulimia nervosa, and OED.

**Table 3:** Economic cost of eating disorders in 2015

	Eating disorders			Anorexia nervosa			Bulimia nervosa			Other eating disorders		
Classification	Male	Female	Sub total	Male	Female	Sub total	Male	Female	Sub total	Male	Female	Sub total
Direct costs												
Direct medical costs	246,792	2,572,075	2,818,867	91,585	1,037,569	1129 154	31,420	737,817	769,237	123,786	796,690	920,476
Direct non-medical costs												
Transportation cost for hospital visits	11,492	84,283	95,776	4402	20,806	25,208	1275	27,047	28,322	5815	36,430	42,245
Caregiver cost	79,798	405,167	484,965	44,865	213,194	258,059	5964	67,603	73,568	28,969	124,370	153,339
Total direct costs	338,082	3,061,526	3,399,608	140,852	1,271,569	1,412,421	38,660	832,467	871,127	158,570	957,490	1,116,060
Indirect costs-2	453,177	1,602,841	2,056,018	132,185	353,510	485,694	18,772	328,218	346,991	302,220	921,113	1,223,333
Total costs	791,259	4,664,367	5,455,626	273,037	1,625,078	1,898,115	57,432	1,160,686	1,218,118	460,790	1,878,602	2,339,393

*Note*. Exchange rate US dollar: 1 Korean won = 1132 US dollar from the Bank of Korea (http://ecos.bok.or.kr/); For indirect costs-2, productivity loss from the absence from work due to hospital admission and outpatient visits were included

Table 4 shows the results of the sensitivity analysis for the economic burden of eating disorders in 2015. OED were the highest contributor to the economic burden and females were a higher contributor to the economic burden than males in indirect costs-1.

**Table 4:** Sensitivity analysis of indirect costs for economic cost of eating disorders in 2015

	Eating disorders			Anorexia nervosa			Bulimia nervosa			Other eating disorders		
Classification	Male	Female	Sub total	Male	Female	Sub total	Male	Female	Sub total	Male	Female	Sub total
Indirect costs-1	920,012	3,064,617	3,984,629	157,353	638,689	796,043	24,599	552,534	577,132	738,060	1,873,394	2,611,454

*Note*. Indirect costs-1 is different from indirect costs-2. Indirect costs-1 was estimated for the purpose of sensitivity analysis without the employment-to-population ratio (i.e., proportion of the population employed). Indirect costs-1 was not included in the total costs

**Fig. 2:**
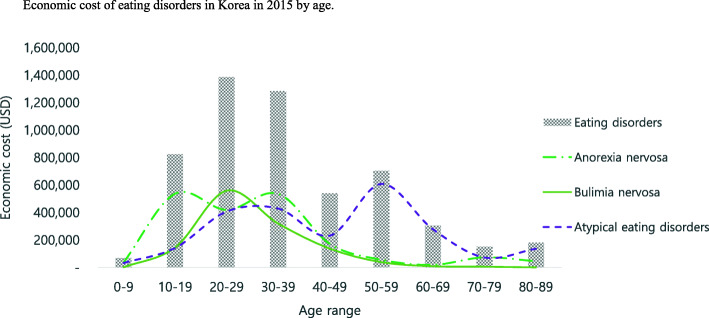
Economic burden of eating disorders in Korea in 2015 by age

**Fig. 3:**
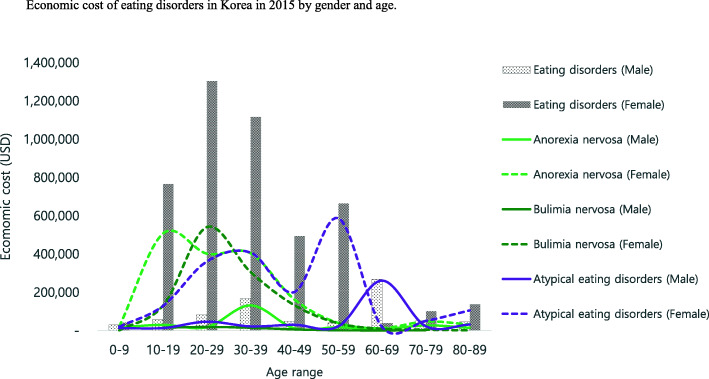
Economic burden of eating disorders in Korea in 2015 by gender and age

Table 5 and Figs. 2 and 3 show the economic burden of eating disorders in Korea in 2015 by age and gender. The economic burden of eating disorders was higher in patients aged between 20 years and 29 years than other age ranges. Anorexia nervosa was higher in patients aged between 10 years and 19 years than other age ranges. Bulimia nervosa was higher in patients aged between 20 years and 29 years than other age ranges. OED were higher in patients aged 50 years and 59 years than other age ranges. In general, female patients showed higher economic burden than male patients. In addition, younger generations showed a higher economic burden than older generations, except for in the case of OED.

**Table 5:** Economic cost of disease due to eating disorders in Korea in 2015 by age group

	Eating disorders						Anorexia nervosa						Bulimia nervosa						Other eating disorders					
	Direct cost			Indirect cost			Direct cost			Indirect cost			Direct cost			Indirect cost			Direct cost			Indirect cost		
Age range	Male	Female	Sub total	Male	Female	Sub total	Male	Female	Sub total	Male	Female	Sub total	Male	Female	Sub total	Male	Female	Sub total	Male	Female	Sub total	Male	Female	Sub total
0–9	29,659	41,561	71,220	–	–	–	16,507	20,429	36,936	–	–	–	–	1253	1253	–	–	–	13,152	19,879	33,031	–	–	–
10–19	58,076	766,065	824,141	–	–	–	29,904	507,441	537,345	–	–	–	15,145	130,128	145,273	–	–	–	13,027	128,496	141,523	–	–	–
20–29	59,903	913,172	973,075	22,652	390,672	413,324	12,388	274,955	287,343	5134	126,763	131,897	13,288	388,018	401,306	5231	152,803	158,034	34,227	250,199	284,427	12,287	111,105	123,392
30–39	42,248	688,260	730,507	126,348	427,966	554,314	26,783	237,700	264,483	105,300	166,309	271,609	6174	198,800	204,974	8619	106,144	114,763	9290	251,760	261,050	12,429	155,513	167,941
40–49	16,621	293,279	309,900	30,714	200,841	231,555	4100	118,678	122,778	7081	42,819	49,901	2130	75,690	77,820	3705	56,011	59,716	10,391	98,911	109,302	19,927	102,011	121,938
50–59	16,330	89,894	106,224	23,474	574,452	597,925	7170	25,813	32,984	10,143	13,574	23,717	286	25,356	25,641	1014	12,263	13,277	8874	38,725	47,599	12,317	548,614	560,931
60–69	27,404	34,994	62,398	240,666	4301	244,967	5041	10,934	15,975	1894	1321	3215	19	8126	8145	27	892	919	22,344	15,934	38,278	238,745	2088	240,833
70–79	47,007	98,324	145,331	4483	3213	7697	24,946	45,507	70,453	2544	1555	4099	1618	4200	5818	176	103	279	20,444	48,617	69,061	1764	1555	3319
80–89	40,834	135,978	176,812	4840	1395	6236	14,013	30,113	44,125	88	1167	1255	–	897	897	–	1	1	26,821	104,968	131,790	4752	227	4979
Total	338,082	3,061,526	3,399,608	453,177	1,602,841	2,056,018	140,852	1,271,569	1,412,421	132,185	353,510	485,694	38,660	832,467	871,127	18,772	328,218	346,991	158,570	957,490	1,116,060	302,220	921,113	1,223,333

*Note*. Exchange rate US dollar: 1 Korean won = 1132 US dollar from the Bank of Korea (http://ecos.bok.or.kr/)

## Discussion

Population-representative epidemiological research studies on eating disorders are rare. Despite the knowledge that eating disorders have an early onset, few studies have been conducted on eating disorders among children and young people under the age of 18 [25]. The current study is meaningful, in that its use of a nationwide database means that it represents all of South Korea, including patients of all ages. It included eating disorder with ICD F50.x in its entirety and was not limited to anorexia nervosa and bulimia nervosa alone.

The recent systematic review reported that the estimated lifetime prevalence of eating disorder was 1.01% (95% CI, 0.54–1.89) [26]. It is noteworthy that the lifetime prevalence reported from studies conducted in Western countries (1.29%) was 6.1-fold greater than that reported in a single study from South Korea (0.21%) [26]. The current study found that the prevalence of eating disorders in South Korea was between 12.02 (0.012%) in 2010 and 13.28 (0.013%)in 2015. This implies that it can update the prevalence of eating disorders in South Korea, even though our study method and case definition varied from that of Cho et al. [22]. The estimated total economic cost of eating disorders in the current study was USD 5455626, which is equivalent to 0.0039% of Korean GDP in 2015. Those with OED, including binge eating disorder, accounted for 42% of the economic burden; anorexia nervosa, 34.7%; and bulimia nervosa, 22.3%. Our results are underestimated because the study did not take into account the negative impact of eating disorders on individual health, or socio-economic well-being. Given this, the actual economic costs can be expected to be much higher. In terms of gender, the treatment prevalence of eating disorders among females was high (4.68–5.27 times) in our study, and the medical expenditure for females was more than twice as high (8.33–12.26 times), compared to the treatment prevalence. In addition, in the proportion of economic burden, the ratio of direct medical cost is significantly higher for women compared to men (55% vs 31%). This is thought to be, in part, due to general gender differences in seeking diagnostic evaluation or healthcare treatment, and receiving more prescription drugs [27–29]. As shown by previous studies, the current study found that the disease burden of eating disorders was high in adolescent and early adult ages. This implies that disease burden is likely underestimated, because it is a condition that can be chronic and progressive [30].

A few limitations in the present study must be noted. First, the data was collected from a secondary database, the NHIS claims database, and not from medical records. It considers only the burden of disease based on patients who sought treatment. Also, we did not consider either psychiatric or physical comorbid disorders. Therefore, questions about the validity of the diagnosis and comorbidity information across hospitals may be raised. In addition, we used the number of hospitalizations and frequency of outpatient visits to ensure accuracy. Another limitation is that binge eating disorder, which has of clinical importance was added to the DSM-5 in 2013, and was not reflected in the ICD diagnostic system during the study period; therefore in our study, it is included under unspecified eating disorders. Although, we used the nationally representative database (i.e. HIRA), the treatment prevalence rates may not represent patients with eating disorders of South Korea, due to the nature of the database using medical records. Thus, future research may replicate this study by assessing another database to calculate the prevalence rates of South Korea.

## Conclusion

Despite these limitations, this study is meaningful in that it has calculated the treatment prevalence and economic burden of eating disorders using national representative data. Eating disorders create severe and disabling conditions for the afflicted individual, their families, and society at large, but are often overlooked. In particular, this study is unique in its inclusion of other eating disorder groups, including binge eating disorder; most previous studies examined only bulimia nervosa and anorexia nervosa. The findings from the current study contribute to the evidence base from which suggestions for improvements in health service can be made, and to make policy- and service-planning more effective.

## Data Availability

No additional data available.

## Abbreviations

DSM: Diagnostic and Statistical Manual
HIRA: Health Insurance Review & Assessment Service
NHIS: National Health Insurance Services
NHID: National Health Information Database
KOSIS: Korean Statistical Information Service
ICD: International Classification of Diseases
OED: other eating disorders

## References

[1] Kessler RC (2013). The prevalence and correlates of binge eating disorder in the World Health Organization world mental health surveys. Biol Psychiatry.

[2] Micali N, et al. The incidence of eating disorders in the UK in 2000–2009: findings from the General Practice Research Database. BMJ Open. 2013;3:e002646. 10.1136/bmjopen-2013-002646. 10.1136/bmjopen-2013-002646 PMC3657659 23793681

[3] Smink FR, van Hoeken D, Hoek HW (2013). Epidemiology, course, and outcome of eating disorders. Curr Opin Psychiatry.

[4] Jacobi F (2004). Prevalence, co-morbidity and correlates of mental disorders in the general population: results from the German health interview and examination survey (GHS). Psychol Med.

[5] Hudson JI (2007). The prevalence and correlates of eating disorders in the National Comorbidity Survey Replication. Biol Psychiatry.

[6] Schmidt U (2016). Eating disorders: the big issue. Lancet Psychiatry.

[7] Lewinsohn PM, Striegel-Moore RH, Seeley JR (2000). Epidemiology and natural course of eating disorders in young women from adolescence to young adulthood. J Am Acad Child Adolesc Psychiatry.

[8] Coomber K, King RM (2013). A longitudinal examination of burden and psychological distress in carers of people with an eating disorder. Soc Psychiatry Psychiatr Epidemiol.

[9] Steinhausen HC (2002). The outcome of anorexia nervosa in the 20th century. Am J Psychiatry.

[10] Sullivan PF (1995). Mortality in anorexia nervosa. Am J Psychiatry.

[11] Zipfel S (2015). Anorexia nervosa: aetiology, assessment, and treatment. Lancet Psychiatry.

[12] Tseng MM (2020). Rates and trends in healthcare-detected incidence of anorexia nervosa and bulimia nervosa: a national health insurance claim data study in Taiwan, 2002-2013. Int J Eat Disord.

[13] Chua SN (2021). Estimated prevalence of eating disorders in Singapore. Int J Eat Disord.

[14] Treasure J (2015). Anorexia nervosa. Nat Rev Dis Primers.

[15] National Collaborating Centre for Mental Health (UK). Eating disorders: core interventions in the treatment and management of anorexia nervosa, bulimia nervosa and related eating disorders. Leicester: British Psychological Society (UK); 2004. 23346610

[16] Zabala MJ, Macdonald P, Treasure J (2009). Appraisal of caregiving burden, expressed emotion and psychological distress in families of people with eating disorders: a systematic review. Eur Eat Disord Rev.

[17] Erskine HE, Whiteford HA, Pike KM (2016). The global burden of eating disorders. Curr Opin Psychiatry.

[18] Gustavsson A (2011). Cost of disorders of the brain in Europe 2010. Eur Neuropsychopharmacol.

[19] Yao S (2021). Screen-detected disordered eating and related traits in a large population sample of females in mainland China: China health and nutrition survey. Int J Eat Disord.

[20] Lee CK (1990). Psychiatric epidemiology in Korea. Part I: gender and age differences in Seoul. J Nerv Ment Dis.

[21] Lee CK (1990). Psychiatric epidemiology in Korea. Part II: urban and rural differences. J Nerv Ment Dis.

[22] Cho MJ (2007). Lifetime and 12-month prevalence of DSM-IV psychiatric disorders among Korean adults. J Nerv Ment Dis.

[23] Cheol Seong S (2017). Data resource profile: the national health information database of the National Health Insurance Service in South Korea. Int J Epidemiol.

[24] World Health Organization. The ICD-10 classification of mental and behavioural disorders: clinical descriptions and diagnostic guidelines. Geneva: World Health Organization; 1992.

[25] Erskine HE (2017). The global coverage of prevalence data for mental disorders in children and adolescents. Epidemiol Psychiatr Sci.

[26] Qian J (2013). Prevalence of eating disorders in the general population: a systematic review. Shanghai Arch Psychiatry.

[27] Anthony M (2008). Gender and age differences in medications dispensed from a national chain drugstore. J Women's Health (Larchmt).

[28] Owens GM (2008). Gender differences in health care expenditures, resource utilization, and quality of care. J Manag Care Pharm.

[29] Schappert SM, Burt CW (2006). Ambulatory care visits to physician offices, hospital outpatient departments, and emergency departments: United States, 2001–02. Vital Health Stat.

[30] Franko DL (2013). A longitudinal investigation of mortality in anorexia nervosa and bulimia nervosa. Am J Psychiatry.

